# Long‐term stability analysis of beam shape in a robotic radiosurgery system

**DOI:** 10.1002/acm2.70123

**Published:** 2025-06-23

**Authors:** Ryoichi Hinoto, Shiho Kashiyama, Takahisa Eriguchi, Nobuhiro Tsukamoto, Takeji Sakae

**Affiliations:** ^1^ Department of Radiation Oncology Saitama Red Cross Hospital Saitama Japan; ^2^ Department of Radiation Oncology Saitama Municipal Hospital Saitama Japan; ^3^ Faculty of Medicine University of Tsukuba Tsukuba Japan

**Keywords:** CyberKnife, flatness, long‐term analysis, penumbra, symmetry

## Abstract

**Purpose:**

This study aimed to investigate the long‐term stability of CyberKnife beam profile parameters and assess their compliance with existing quality assurance (QA) guidelines. We evaluated beam profiles in both standard and diagonal planes over 3.5 years post‐installation to detect potential issues and ensure consistent beam quality. The findings will contribute to validating and refining current QA practices for CyberKnife systems.

**Methods:**

Beam profile measurements were performed monthly using an Octavius 1000SRS detector array. The profiles were evaluated in terms of the beam shape constancy within 2%, and the penumbra, symmetry, and flatness were analyzed using statistical process control methods. Temporal changes in the dose profiles were visualized using dose difference heat maps. The relationship between the beam parameters and accumulated monitor units was also investigated.

**Results:**

The 2% profile constancy check accurately detected magnetron deterioration 2 months before failure, confirming its high sensitivity for beam stability monitoring. While symmetry and flatness remained within 0.7% throughout over 100 × 10^6^ monitor units of operation, penumbra exhibited greater responsiveness to magnetron‐induced changes but did not consistently flag all orientations. Additionally, statistical analyses and heat maps revealed gradual profile shifts independent of acute component failures, highlighting the importance of multifaceted QA strategies.

**Conclusions:**

These findings reinforce the effectiveness of the 2% profile constancy check for early detection of magnetron failure and support its adoption in current CyberKnife guidelines. At the same time, symmetry, flatness, and penumbra parameters remain valuable for characterizing gradual profile variations. Collectively, this study underscores the need for comprehensive beam monitoring and regular maintenance to sustain optimal CyberKnife system performance.

## 1 INTRODUCTION

The CyberKnife system is a robotic radiosurgery platform that delivers highly precise radiation treatments.^1^ Ensuring the long‐term stability and consistency of the CyberKnife beam profiles is crucial for maintaining treatment quality and patient safety. Despite this importance, comprehensive studies examining the temporal changes in CyberKnife beam profiles over extended periods are lacking.

This study presents the first long‐term analysis of the CyberKnife beam profile stability by investigating the presence of time‐dependent variations. Existing quality assurance (QA) guidelines for CyberKnife systems, such as the original report of AAPM TG‐135,^2^ the recently updated TG‐135.B report,^3^ and the Canadian Partnership for Quality Radiotherapy (CPQR) technical quality control guidelines,^4^ include criteria for beam symmetry but do not provide specific recommendations for the flatness or penumbra distance. In flattening filter free (FFF) beams, the change in the central peak is pronounced due to variations in beam energy, and the change in the flatness parameter is more sensitive than the change in percentage depth dose (PDD).^5^ In contrast, the AAPM TG‐142 report,^6^ which focuses on QA for C‐arm linear accelerators used in conventional radiation therapy, includes flatness criteria. Similarly, the penumbra is also highly sensitive to beam energy variations, as energy variation can substantially alter the penumbra characteristics.^7^, ^8^ Stereotactic radiosurgery (SRS) and stereotactic radiotherapy (SRT) treatments require a steep penumbra to protect the normal tissue around the tumor.^8^, ^9^, ^10^, ^11^ This sensitivity of both flatness and penumbra to energy variations in FFF beams underscores the importance of establishing specific QA recommendations for these parameters.

To address these gaps, we conducted a comprehensive study of CyberKnife beam profiles over a period of approximately 4 years after installation. By analyzing the profiles in both the standard transverse and diagonal orientations, we aimed to detect abnormal values or trends that could indicate component deterioration or impending failure. Therefore, the results of this study provide valuable insight into the long‐term performance of CyberKnife systems and highlight the importance of regular monitoring and maintenance to ensure consistent beam quality over time.

This study aims to contribute to the refinement of QA practices for CyberKnife systems by providing a comprehensive analysis of the long‐term beam profile. Our findings may inform future updates to the QA guidelines and ultimately enhance the consistency and quality of SRS and SRT using the CyberKnife platform.

## 2 METHODS

### 2.1 Data collection

**FIGURE 1:**
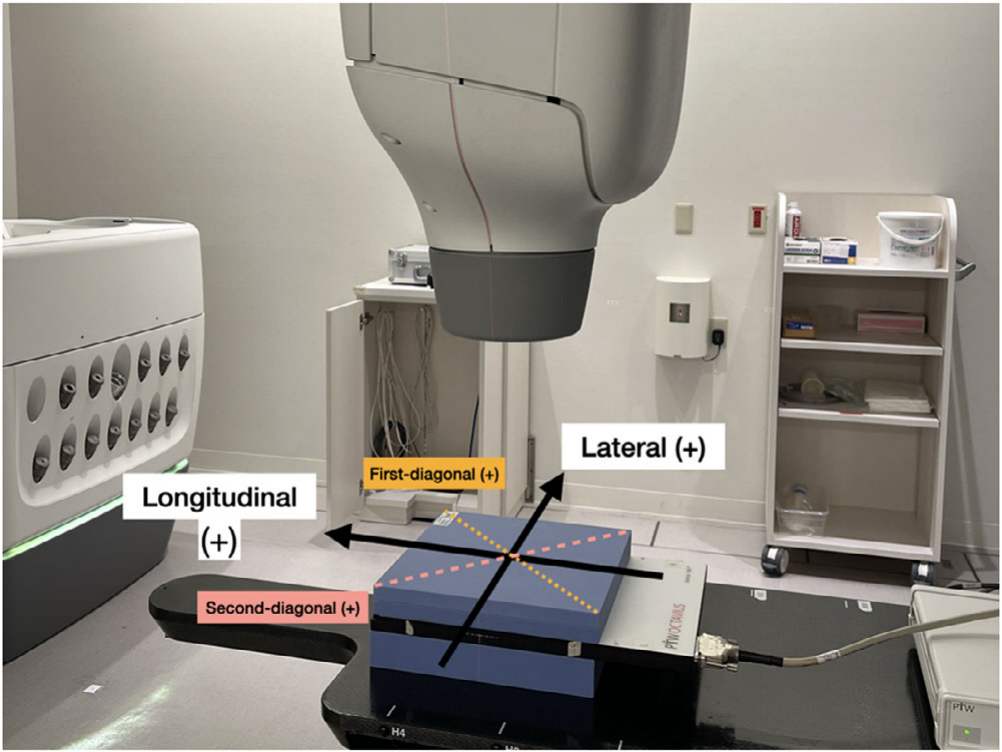
Schematic diagram of the measurement setup. The figure shows the arrangement of the Octavius 1000SRS detector array and Solid Water HE relative to the CyberKnife beam. The scanning plane is also illustrated to demonstrate the profile measurement orientation.

Beam profile measurements were performed using a CyberKnife M6 (Accuray Inc., Madison, WI, USA) at an SSD of 74.9 cm and SAD of 80 cm with an Octavius 1000SRS detector array (PTW‐Freiburg, Germany). The Octavius 1000SRS features 977 liquid‐filled ionization chambers arranged in a square configuration, with a high‐resolution central area and a larger outer region. This compact detector array is well‐suited for small‐field dosimetry and has been extensively evaluated for use with various linear accelerators, including CyberKnife.^12^, ^13^, ^14^ The detector array was set up with an inherent water‐equivalent layer and additional solid water HEs (Sun Nuclear Corporation, Madison, WI, USA) placed above and below it to account for backscatter effects (Figure 1). It was initially positioned using the room‐mounted lasers, and then the CyberKnife centerline laser was aligned with the detector's center by adjusting the robotic arm. A 60 mm fixed collimator was used, and 100 monitor units (MU) were delivered to measure the beam profiles. The initial measurement served as the baseline profile, with subsequent measurements performed monthly from February 2021 to July 2024. To minimize measurement uncertainty, the baseline profile was established as the average of five repeated measurements.

To investigate the relationship between the beam profile and the total MU used, we obtained the cumulative MU, referred to as the elapsed MU, on each measurement date. The elapsed MU was determined by selecting the baseline measurement date and each monthly check date from “Report Administration—System—CyberKnife System” in Precision version 3.3.1.3 and referring to the “Monitor Units” in the output report.

Approximately 1.5 years after installation, failure occurred owing to magnetron damage, necessitating replacement. At the time of failure, the elapsed MU value was approximately 72 × 10^6^. Following the magnetron replacement, beam profile adjustments were performed by adjusting the steering coil current.

The measurement data saved by the Octavius system were stored as text files. In this study, we investigated long‐term changes in the CyberKnife beam profile using these text files. The handling of the textual data is described in detail in the following sections.

### 2.2 Evaluation of beam shape

In TG‐135, a monthly check for the beam shape requires that it remains within 2% of the baseline.^2^ To evaluate whether the CyberKnife system meets these criteria over an extended period, we calculated the pass rates for dose profiles in the longitudinal, lateral, first diagonal, and second diagonal directions using the respective criteria, with the profiles at installation serving as the baseline. Pass rates were evaluated based on data points where the dose level exceeded 10% of the maximum dose in each profile.

To eliminate minor deviations in the central axis (CAX_center_) position of each profile, the full width at half maximum (FWHM) was calculated from the profile data to determine the center, which is referred to as the CAX_center_. Each dataset was then linearly interpolated to generate coordinates at every 0.01 mm centered around the CAX_center_.

### 2.3 Evaluation of penumbra, symmetry, and flatness

The key parameters analyzed were the penumbra width on both sides, symmetry, and flatness. Although TG‐135 specifies a criterion of within 3% for symmetry,^2^ it does not specify criteria for the flatness or penumbra. Moreover, although TG‐142 provides a 1% criterion for flatness in annual checks for C‐arm linear accelerators,^6^ this is not specified for the CyberKnife systems in TG‐135.

The data for these parameters were obtained for four beam orientations: longitudinal, lateral, first diagonal, and second diagonal. These data were processed using the same methods applied in Section 2.2, “Evaluation of Beam Shape.”

**Penumbra**

The penumbra width was defined as the distance between the 80% and 20% dose points in the relative dose profile. This region represents the transitional area between the high and low dose regions of the radiation beam, and it is a key factor in assessing the beam sharpness and edge gradient in SRS and SRT.^8^, ^9^, ^10^, ^11^

**Symmetry**

The symmetry was calculated according to the IEC 60976 standard. Symmetry, *S*, is the maximum value of the ratio of the higher to lower absorbed dose at any two positions, denoted by *x* and −*x*, which are symmetric relative to the CAX_center_ within the central 80% of the FWHM of the beam:

$$S=\max\left(\frac{\max(D(x),\;D(-x))}{\min(D(x),\;D(-x))}\right)\times 100$$

Here, *x* corresponds to the position in each scanned plane (longitudinal, lateral, first diagonal, second diagonal). This parameter quantifies how uniformly the beam delivers a dose across symmetrically opposite points, which is critical for ensuring an even dose distribution in treatments.

**Flatness**

The flatness was determined according to the IEC 60976 standard. The flatness *F* is defined as the ratio of the maximum absorbed dose to the minimum absorbed dose within the central 80% of the FWHM of the beam:

$$F=\frac{D_{\max}}{D_{\min}}\times 100$$

The flatness assesses how consistently the dose is distributed across the central portion of the beam, ensuring that the dose remains uniform across the treatment field.

Although the IEC 60976 standard defines an alternative method for calculating the symmetry and flatness of diagonal profiles, this study employed the aforementioned calculation method because the diagonal profiles analyzed are derived from circular collimator data.

#### 2.3.1 Statistical analysis methods

The beam data were analyzed using Shewhart control charts,^15^, ^16^ supplemented by moving average analysis^17^ and Mann–Kendall trend testing^18^ to assess the stability and presence of trends in the data.

**Shewhart Control Charts**

Shewhart control charts were constructed by calculating the mean and standard deviation (*σ*) for each parameter, based on data collected from the machine's installation until immediately before the occurrence of a failure. This period covered approximately 1.5 years, with an elapsed MU of approximately 72 × 10^6^. The upper control limit (UCL) and lower control limit (LCL) were set at ± 3*σ* from the mean to provide boundaries for identifying significant deviations in the beam parameters over time.

**Moving Average Analysis**

A moving average with a window size of 10 was applied to the data and plotted to highlight gradual trends that might not be immediately apparent from the raw data points. This method aids in visualizing subtle changes over time, which could indicate shifts in beam stability.

**Mann–Kendall Trend Test**

The Mann–Kendall trend test, which is a nonparametric statistical tool, was utilized to detect monotonic trends in the beam parameters over time. That is, the test is well‐suited for identifying consistent upward or downward trends and does not require the data to follow a specific distribution. Hence, this test is ideal for quality control data that may exhibit non‐normal characteristics or include outliers.

### 2.4 Temporal changes in dose profiles

For each monthly check date corresponding to the elapsed MU, we calculated the dose difference between the profile at that date and the baseline profile. Dose differences were calculated for dose levels greater than 10% of the maximum dose in each profile. The data for these profiles were processed using the same methods applied in Section 2.2, “Evaluation of Beam Shape.”

To visualize the changes in the beam profiles relative to the baseline over time, we generated time‐series heat maps of the dose differences. These heat maps provide an intuitive representation of the evolution of beam profiles with accumulated usage to enable the identification of trends or abnormal values.

To further investigate beam characteristics over time, we performed a two‐dimensional (2D) dose profile analysis in addition to the one‐dimensional profile analysis. For each monthly check date, we calculated the dose difference between the 2D dose profile and the baseline profile. These dose differences were averaged across all data to visualize the mean dose change in the beam profile since installation. The mean dose difference data were visualized using heat maps, providing insights into the spatial distribution of changes in the beam profile over time.

Two‐dimensional dose profiles were generated from the text data recorded by the Octavius system during measurements. Based on the coordinates of the CAX_center_, determined from the longitudinal and lateral profiles, we located CAX_center_ in the 2D dose distribution. We then performed 2D linear interpolation on data from four orientations – longitudinal, lateral, first diagonal, and second diagonal – to obtain a dose map with a resolution of 0.2 mm × 0.2 mm. Dose differences were calculated for dose levels greater than 50% of the maximum dose in each profile, focusing on the central high‐dose region of the beam. For the analysis of mean dose differences, data corresponding to the period when the 2% dose difference pass rate fell below 90% were excluded to focus on beam profile changes unrelated to the magnetron failure.

## 3 RESULTS

### 3.1 Evaluation of beam shape

**FIGURE 2:**
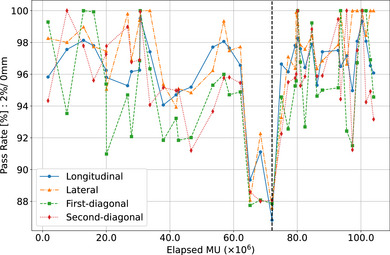
Pass rates for TG‐135 criteria (2% dose difference) compared to baseline over time. The graph shows the results for longitudinal, lateral, first diagonal, and second diagonal orientations. The black dashed line indicates the magnetron replacement.

Figure 2 presents the pass rates for a 2% dose difference criterion compared to the baseline over time for the CyberKnife system. The graph shows the results for each monthly check across the four measured orientations: longitudinal, lateral, first diagonal, and second diagonal. A vertical black dashed line indicates the point at which measurements were taken immediately following the replacement of the magnetron.

The graph reveals distinct patterns in beam orientations. For the longitudinal and lateral orientations, the dose difference pass rates remained above 94% throughout most of the observation period. In contrast, the first diagonal and second diagonal orientations exhibited slightly lower pass rates but still maintained levels above 90% for the majority of the time.

Approximately 1 to 2 months before the magnetron failure – indicated by the black dashed line – the pass rates dropped below 90%. After the magnetron was replaced and the magnetron current and steering coil current were adjusted, the pass rates returned to levels comparable to those before the failure.

### 3.2 Evaluation of penumbra, symmetry, and flatness

**FIGURE 3:**
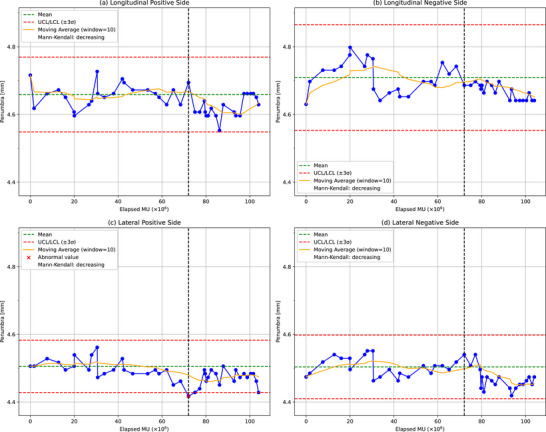
Penumbra measurements for both sides over time for longitudinal and lateral orientations. (a) Longitudinal positive side, (b) longitudinal negative side, (c) lateral positive side, (d) lateral negative side. The black dashed line indicates the magnetron replacement. LCL, lower control limit; UCL, upper control limit.

**FIGURE 4:**
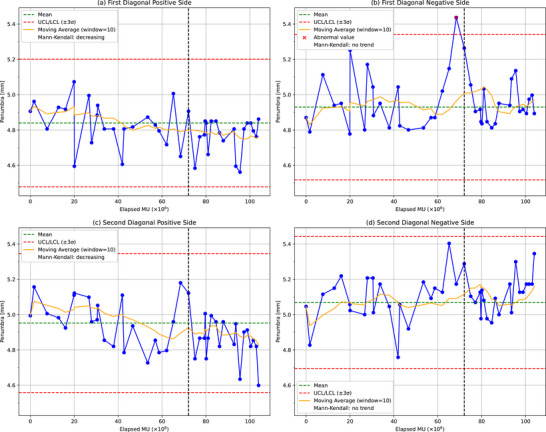
Penumbra measurements for both sides over time for first diagonal orientation. (a) First Diagonal Positive Side, (b) First Diagonal Negative Side, (c) Second Diagonal Positive Side, (d) Second Diagonal Negative Side. The black dashed line indicates the magnetron replacement. UCL: upper control limit, LCL: lower control limit.

**FIGURE 5:**
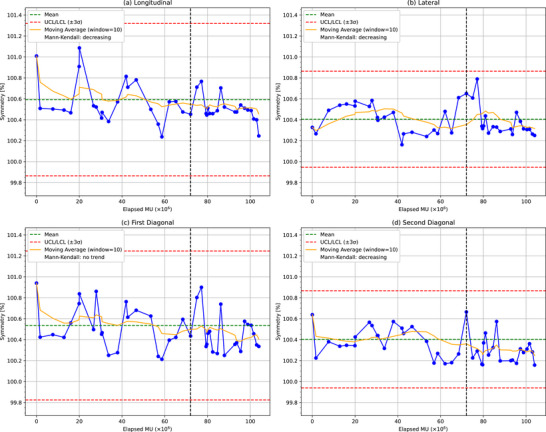
Symmetry measurements over time for (a) longitudinal, (b) lateral, (c) first diagonal, and (d) second diagonal orientations. The black dashed line indicates the magnetron replacement. UCL: upper control limit, LCL: lower control limit.

**FIGURE 6:**
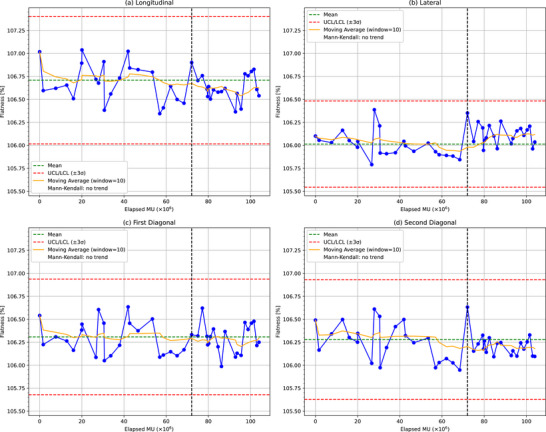
Flatness measurements over time for (a) longitudinal, (b) lateral, (c) first diagonal, and (d) second diagonal orientations. The black dashed line indicates the magnetron replacement. UCL: upper control limit, LCL: lower control limit.

Figures 3, 4, 5, 6 present the analytical results for the penumbra (on both sides), symmetry, and flatness across the four beam profiles: longitudinal, lateral, first diagonal, and second diagonal. Each graph includes control limits derived from Shewhart control charts based on data collected up to 2 months prior to the magnetron replacement, with abnormal values indicated by red X marks. These control limits were established considering the measurement uncertainties, which were evaluated by repeating the baseline measurements five times. The standard deviations of these repeated measurements were 0.65% for the penumbra and 0.3% for the symmetry and flatness. The solid yellow line represents the moving average over the elapsed MU to facilitate the visualization of trends in the parameters.

1. Penumbra: In the longitudinal orientation (Figure 3a,b), both sides of the penumbra showed a significant decreasing trend that was particularly pronounced in the negative side and exhibited a highly significant decrease over time (*p* < 0.001), as shown in Table 1. No abnormal values were detected before the magnetron replacement. For the lateral orientation (Figure 3c,d), an abnormal value was detected on the positive side as the magnetron was replaced. Both sides demonstrated significant decreasing trends (*p* < 0.001) throughout the observation period, with the positive side showing a significant decrease even before failure (*p* = 0.006), as listed in Table 1.

**TABLE 1:** Penumbra trend analysis: Longitudinal and lateral.

Orientations	Full period	Before failure	After failure
Longitudinal positive	↓ (*p* = 0.029)	NS	NS
Longitudinal negative	↓ (*p* < 0.001)	NS	↓ (*p* = 0.001)
Lateral positive	↓ (*p* < 0.001)	↓ (*p* = 0.006)	NS
Lateral negative	↓ (*p* < 0.001)	NS	NS

*Note*: Trends were determined using Mann–Kendall Trend Test. Downward arrows (↓) decreasing trends, and NS (No Significant trend) indicates no trend was detected. “Failure” refers to magnetron failure.

In the first diagonal orientation (Figure 4a,b), the positive side showed a significant decrease (*p* = 0.032), whereas no trend was observed on the negative side, as shown in Table 2. An abnormal value was detected on the negative side 1 month before the magnetron replacement. No abnormal values were detected in the second diagonal orientation (Figure 4c,d). The positive side demonstrated a highly significant decrease (*p* = 0.001), with a significant decreasing trend before failure (*p* = 0.032), whereas no such trend was observed on the negative side, as listed in Table 2.

**TABLE 2:** Penumbra trend analysis: first and second diagonal.

Orientations	Full period	Before failure	After failure
First diagonal positive	↓ (*p* = 0.032)	NS	NS
First diagonal negative	NS	NS	NS
Second diagonal positive	↓ (*p* = 0.001)	↓ (*p* = 0.032)	NS
Second diagonal negative	NS	NS	NS

*Note*: Trends were determined using Mann–Kendall Trend Test. Downward arrows (↓) decreasing trends, and NS (No Significant trend) indicates no trend was detected. “Failure” refers to magnetron failure.

Overall, the data indicate that the longitudinal and lateral orientations exhibit lower measurement variability than do the first and second diagonal orientations. Among the longitudinal and lateral orientations, the negative side of the longitudinal orientation showed the largest variability of *σ* ≈ 0.05 mm, leading to control limits of 0.16 mm (a relative value of 3.3%). For the first diagonal and second diagonal orientations, the negative side of the first diagonal orientation exhibited the largest variability of *σ* ≈ 0.14 mm, resulting in control limits of 0.41 mm (a relative value of 8.4%).

1. Symmetry: No abnormal values were detected in any of the orientations using the Shewhart control charts. As listed in Table 3, significant decreasing trends were observed in all orientations, except for the first diagonal. The longitudinal and lateral orientations showed significant decreasing trends over the full period (*p* = 0.030 and *p* = 0.034, respectively). The lateral orientation also exhibited a significant decreasing trend after the magnetron failure (*p* = 0.001). The second diagonal orientation demonstrated a significant decreasing trend over the full period (*p* = 0.010). The symmetry values for all orientations remained within 1% of the baseline. Furthermore, in absolute terms, these values consistently stayed below the 3% threshold prescribed by TG‐135 throughout the entire observation period. The longitudinal orientation showed the largest data variability of *σ* ≈ 0.24%. The control limit corresponded to 0.7%.
2. Flatness: No abnormal values were detected in any of the orientations using the Shewhart control charts. As shown in Table 4, no significant trends were observed over the full period in any orientation. The lateral orientation exhibited a significant decreasing trend before the magnetron failure (*p* = 0.001), and the second diagonal orientation also showed a significant decreasing trend before failure (*p* = 0.019). No trends were detected in the other orientations or after failure. The longitudinal orientation showed the largest data variability of *σ* ≈ 0.23%. The control limits corresponded to 0.7%.

**TABLE 3:** Symmetry trend analysis.

Orientations	Full period	Before failure	After failure
Longitudinal	↓ (*p* = 0.030)	NS	NS
Lateral	↓ (*p* = 0.034)	NS	↓ (*p* = 0.001)
First diagonal	NS	NS	NS
Second diagonal	↓ (*p* = 0.010)	NS	NS

*Note*: Trends were determined using Mann–Kendall Trend Test. Downward arrows (↓) decreasing trends, and NS (No Significant trend) indicates no trend was detected. “Failure” refers to magnetron failure.

**TABLE 4:** Flatness trend analysis.

Orientations	Full period	Before failure	After failure
Longitudinal	NS	NS	NS
Lateral	NS	↓ (*p* = 0.001)	NS
First diagonal	NS	NS	NS
Second diagonal	NS	↓ (*p* = 0.019)	NS

*Note*: Trends were determined using Mann–Kendall Trend Test. Downward arrows (↓) decreasing trends, and NS (No Significant trend) indicates no trend was detected. “Failure” refers to magnetron failure.

### 3.3 Temporal changes in dose profiles

**FIGURE 7:**
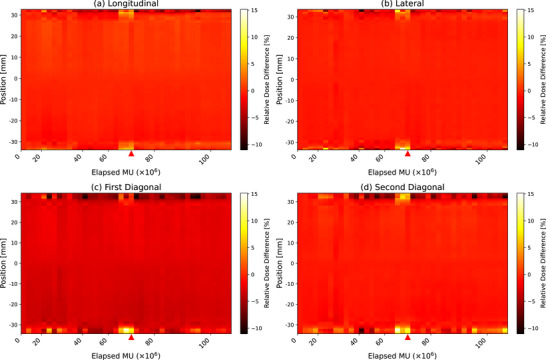
Time series heatmaps of dose profile changes for (a) longitudinal, (b) lateral, (c) first diagonal, and (d) second diagonal orientations. The red triangle indicates the magnetron replacement. The relative dose differences represent the differences between each measurement and the baseline in relative dose.

Figure 7 presents the time‐series heat maps for the four beam orientations: (a) longitudinal, (b) lateral, (c) first diagonal, and (d) second diagonal. These heat maps illustrate the evolution of the dose profiles over time, with the color intensity representing the magnitude of change, relative to the baseline. The red triangle indicates the time of the magnetron failure, before which approximately 2 months, an increase in dose differences within the penumbra regions was observed across all beam orientations.

By organizing the dose difference data into matrices – with each row representing a different elapsed MU and each column corresponding to a spatial position along the profile – we facilitated the continuous visualization of dose profile changes over time. This arrangement reveals both the spatial locations and temporal evolution of the dose changes, which is crucial information that cannot be discerned from dose difference pass rates alone.

**FIGURE 8:**
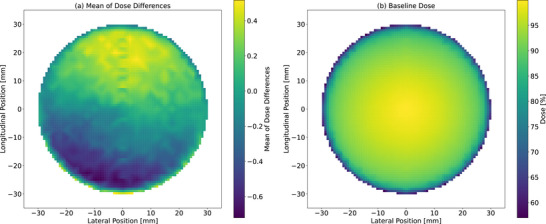
(a) 2D heatmap of the mean of dose differences in beam profiles over time relative to the baseline. The mean of absolute dose differences represents the average of the differences between each monthly measurement and the baseline. (b) 2D heatmap of the baseline dose profiles.

Figure 8 presents a 2D heat map illustrating (a) the mean of the dose differences in the beam profiles over time and (b) the baseline dose profiles. This visualization provides a comprehensive view of how the overall dose distribution shifted throughout the monitoring period. Data corresponding to the period when the 2% dose difference pass rate fell below 90% were excluded from this analysis to focus on beam profile changes unrelated to the magnetron failure.

A distinct directional pattern was observed in the heat map, with an increase in intensity on the positive side and a decrease on the negative side in the longitudinal profile, relative to the baseline. This directional shift in the beam profile provides an insight into how the dose distribution changes over time.

## 4 DISCUSSION

The results of this study demonstrate that while the CyberKnife system generally maintains stable beam profiles over the long term, there are detectable changes in the profiles preceding magnetron failure. These findings extend our understanding beyond the initial beam stability typically observed during system installation and commissioning,^19^ underscoring the importance of regular monitoring to ensure consistent system performance. In addition, the 3.5‐year observation period and an annual caseload of over 550 patients provided a robust dataset to investigate the relationship between beam profile changes and a cumulative usage of over 100 × 10^6^ MU. This intensive utilization also enabled an assessment of the system's long‐term stability.

**FIGURE A1:**
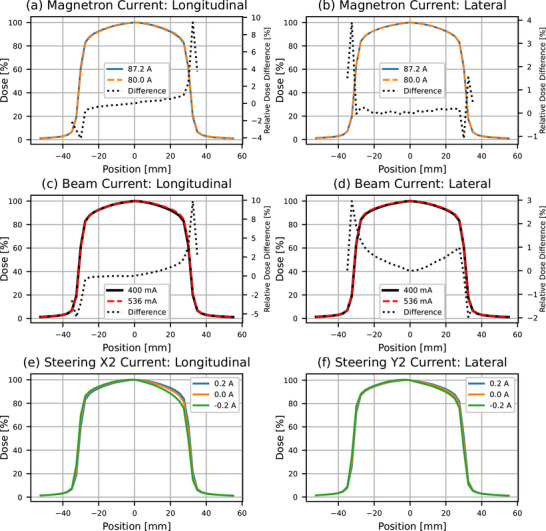
Effects of magnetron current on (a) longitudinal and (b) lateral profiles, beam current on (c) longitudinal and (d) lateral profiles, steering X2 current on (e) longitudinal profile, and steering Y2 current on (f) lateral profile.

**FIGURE A2:**
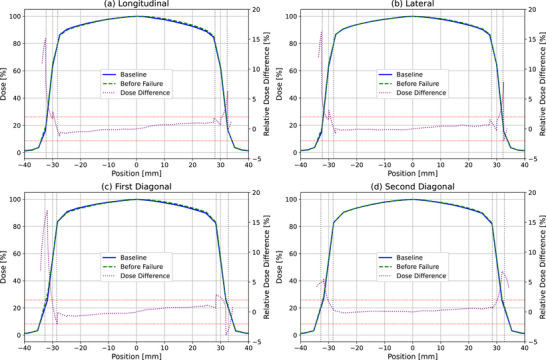
Comparison of beam profiles immediately before magnetron failure (green) and baseline profiles (blue) for (a) longitudinal, (b) lateral, (c) first diagonal, and (d) second diagonal orientations. Black dashed lines mark the 80% and 20% dose levels, red dashed lines indicate the 2% dose difference threshold, and purple dashed lines depict the relative dose differences between the baseline and pre‐failure profiles.

A key outcome of the present study is the 2% constancy check relative to the baseline profile established at installation. As shown in Figure 2, the pass rate dropped below 90% 2 months before the magnetron failure, indicating a substantial deviation in beam profiles. After magnetron replacement, adjustments to the steering coil current and magnetron current restored the profiles closer to the original baseline. The effects of these current adjustments on the beam profile trends are shown in Appendix Figure A1. To elucidate how the declining pass rate corresponds to changes in beam profiles, we employed a time‐series heat map (Figure 7). Red triangles mark the magnetron replacement (aligned with the dashed black line in Figure 2), but significant differences in the penumbra region had already emerged 2 months earlier. Appendix Figure A2 further illustrates beam profiles just prior to failure compared with baseline profiles in each orientation, revealing that magnetron‐related issues have minimal impact inside the 80% dose region yet prominently affect the penumbra region. The magnetron, which generates the microwave power for electron acceleration, is critical to maintaining consistent beam energy in the linear accelerator. As it deteriorates, insufficient microwave power leads to decreased electron acceleration and consequently lower beam energy. While the beam center (inside the 80% region) shows minimal change, this energy deficit substantially alters the penumbra (Appendix Figure A1). Similar findings on the penumbra's sensitivity to beam energy variations have been reported by Thomas^7^ and O'Malley et al.^8^

The findings presented in the previous paragraph also facilitate the interpretation of the long‐term observations of penumbra, symmetry, and flatness. Notably, even at magnetron failure, neither symmetry nor flatness exhibited abnormal values on the Shewhart control charts. This suggests that symmetry and flatness are less sensitive to magnetron‐induced perturbations, as both parameters are predominantly calculated over the 80% dose region of the FWHM, which largely overlaps with, although not identical to, the within 80% dose region of the beam profile where the effects of magnetron failure are less apparent. In contrast, penumbra parameters recorded abnormal values around the time of magnetron replacement, particularly in the lateral positive side (Figure 3c) and the first diagonal negative side (Figure 4b) 1 month before failure. However, while the heat map (Figure 7) reveals relative dose differences in the penumbra regions across all orientations, these dose differences did not consistently translate to abnormal values in the penumbra width measurements for all profiles. As detailed in Appendix Figure A2, the first diagonal negative side showed a dose decrease of 2% at the 80% dose and a 17% increase at the 20% dose, leading to a substantial widening of the penumbra. In contrast, the longitudinal and lateral negative sides exhibited no change at the 80% dose (0%) and a 16% increase at the 20% dose. This indicates that penumbra width can vary significantly when the 20% and 80% dose levels shift in opposite directions but may remain relatively unchanged otherwise. Consequently, although penumbra is more responsive to magnetron‐induced profile changes than symmetry and flatness, it does not consistently demonstrate high sensitivity or a reliable detection capability across all orientations. For this reason, the 2% profile constancy check emerges as the most suitable parameter for identifying magnetron failure in this study.

Focusing on long‐term stability, symmetry and flatness demonstrated remarkable consistency despite cumulative usage exceeding 100 × 10^6^ MU. The Shewhart control limits for these parameters in all directions were consistently within 0.7% of the baseline. This finding suggests that a 1% benchmark for the CyberKnife system is feasible. The results also indicate that CyberKnife may meet the quality control standard of less than 1% for SRS and SRT, as required by the TG‐142 for C‐arm linacs.^6^ Furthermore, the symmetry remained within 3%, the absolute threshold recommended by TG‐135.^2^ Appendix Table A1 presents the control limits for each parameter across various orientations. The Shewhart control limits for penumbra remained within 0.2 mm for longitudinal and lateral profiles and 0.4 mm for diagonal profiles throughout the observation period, excluding the time when magnetron deterioration likely affected the measurements. In SRS and SRT, a narrow penumbra is essential for achieving steep dose gradients near critical structures. Therefore, maintaining a stable penumbra is crucial for minimizing normal tissue exposure.^8^, ^9^, ^10^, ^11^

**FIGURE A3:**
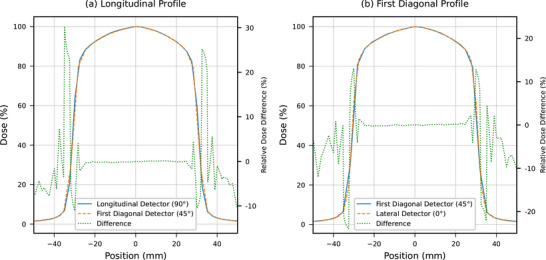
(a) Beam profile differences for the CyberKnife longitudinal beam, measured with the Octavius detector's longitudinal and first‐diagonal channels. (b) Beam profile differences for the CyberKnife first‐diagonal beam, measured with the Octavius detector's first‐diagonal and lateral channels.

Throughout this analysis, evidence emerged suggesting directional dependence in the detector response. Specifically, when the detector array was rotated by 45°, the penumbra width along diagonal detector channels became slightly wider than those along orthogonal channels (longitudinal and lateral), as detailed in Appendix Figure A3. Consequently, the observed differences between diagonal and orthogonal beam components are likely attributable to the inherent directional response of the Octavius detector rather than characteristics of the CyberKnife beam itself. However, when abnormal beam profile values were detected, we performed additional measurements by rotating the detector array by 180° to exclude detector‐dependent effects. The measured profiles remained consistent regardless of detector orientation, suggesting that the observed abnormalities were attributable to the CyberKnife system. Therefore, we conclude that the long‐term stability of the Octavius detector did not significantly influence our analysis results.

The Mann–Kendall trend analysis (Figures 3, 4, 5, 6) revealed significant trends in the penumbra and symmetry for certain orientations, whereas flatness remained stable throughout the observation period. These trends were further corroborated by the 2D heat maps (Figure 8), which highlight directional variations in beam intensity, particularly showing higher intensity on the positive side and lower intensity on the negative side in the longitudinal profile. The robustness of the Mann–Kendall method to outliers strengthens the conclusion that these observed trends reflect genuine long‐term changes rather than temporary fluctuations. Furthermore, since the 2D heat maps exclude data from the period when the 2% dose difference pass rate fell below 90% during the magnetron failure, the observed patterns appear independent of acute component malfunction effects. This comprehensive analysis, combining statistical trend detection and visual representation techniques, reveals subtle beam profile changes that persist beyond the magnetron failure period. The heat maps, by capturing both the magnitude and direction of dose deviations, provide a powerful complementary tool to routine profile measurements, enabling the detection of variations that might otherwise go unnoticed. These findings not only suggest that penumbra and symmetry are more sensitive to gradual changes in beam profiles than flatness but also emphasize the importance of employing multiple analytical approaches for effective long‐term beam monitoring and QA.

While CyberKnife guidelines (TG‐135^2^ and TG‐135B^3^) are shifting from parameters designed for flat beams toward comprehensive beam profile evaluation, our study highlights the 2% profile constancy check as the most sensitive QA indicator. Thus, these findings strongly support the profile constancy check strategy outlined in both TG‐135 and TG‐135B. However, this does not render symmetry, flatness, and penumbra obsolete; these parameters remain relevant for profile changes and, particularly through trend analysis, enable the detection of gradual beam profile variations distinct from acute events such as magnetron failure. The observation of gradual beam profile changes independent of acute component failure emphasizes the importance of comprehensive quality management and regular maintenance procedures for maintaining CyberKnife system performance.

## 5 CONCLUSION

This 3.5‐year study of CyberKnife beam profile shapes, with cumulative usage exceeding 100 × 10^6^ MU, demonstrates three key findings regarding beam stability monitoring. First, the 2% profile constancy check recommended by TG‐135^2^ proved to be a highly sensitive indicator, successfully detecting magnetron deterioration 2 months before failure. This finding validates the shift toward comprehensive profile evaluation in current guidelines. Second, symmetry and flatness measurements consistently remained below 0.7%, demonstrating the feasibility of implementing a 1% tolerance criterion for CyberKnife systems, comparable to the stringent standards established for C‐arm linear accelerators in TG‐142.^6^ Third, excluding periods of magnetron deterioration, penumbra measurements showed remarkable reproducibility, maintaining stability within 0.2 mm for longitudinal and lateral profiles and 0.4 mm for diagonal profiles.

Statistical trend analysis and heat map visualization revealed gradual beam profile changes independent of acute component failures, emphasizing the importance of comprehensive quality management programs. These findings underscore the need for regular maintenance procedures and multiple analytical approaches in the long‐term monitoring of CyberKnife system performance.

## 6 LIMITATIONS

This study focused on the stability of beam profiles and did not assess how changes or abnormal values in these profiles affect clinical dose distributions. In practice, even when the pass rate for the 2% dose difference criteria fell below 90%, patient‐specific QA using the 2%/2 mm criteria outlined in TG‐135^2^ detected no abnormal values.

Another limitation is the reliance on the Octavius system for monitoring CyberKnife profiles. The potential variability of the measurement system warrants further investigation. Additionally, our suggestion for the 1% criterion for symmetry and flatness is based on measurements using the Octavius system, and this criterion might be detector‐dependent. Facilities using different detector systems may need to establish their own appropriate criteria based on their specific measurement uncertainties.

## AUTHOR CONTRIBUTIONS

Ryoichi Hinoto was responsible for the study design, data collection, data analysis, and drafting of the manuscript. Shiho Kashiyama and Takahisa ERIGUCHI critically reviewed the manuscript, providing important feedback for improvement. Nobuhiro Tsukamoto assisted with the organization and structure of the manuscript. Takeji Sakae contributed to the overall review of the manuscript and supported the interpretation of representational data. All authors read and approved the final version of the manuscript.

## CONFLICT OF INTEREST STATEMENT

The authors declare no conflicts of interest.

## ETHIC STATEMENT

This study involved the analysis of machine performance data obtained during routine clinical QA procedures. Inasmuch as no human participants or identifiable patient data were involved, ethical approval was not required.

## Data Availability

The data that support the findings of this study were obtained from routine quality assurance procedures of clinical equipment. These data are not publicly available due to institutional policies but may be available from the corresponding author upon reasonable request.

## EFFECTS OF MAGNETRON CURRENT, BEAM CURRENT, AND STEERING CURRENT ADJUSTMENTS ON BEAM PROFILES

Figure A1 illustrates the change in the beam profile as the magnetron current, beam current, and steering current are varied. The top panel shows the effect of magnetron current on the beam profile, the middle panel demonstrates the influence of beam current, and the bottom panel presents the relationship between the steering current, located downstream of the system, and the beam profile. Each current was varied independently, and the resulting profiles were scanned using the Octavius detector system. Diagonal profiles are omitted. Only the profile in the direction in which the change in steering coil current occurred is shown.

## COMPARISON OF PRE‐FAILURE AND BASELINE BEAM PROFILES

Figure A2 compares the beam profiles immediately prior to magnetron failure with the baseline profiles. The comparison reveals pronounced dose discrepancies in the penumbra regions, particularly on the negative sides, demonstrating the asymmetric impact of magnetron deterioration across all orientations.

## CONTROL LIMITS FOR PROFILE PARAMETERS

Table A1 lists the control limits for each parameter in each profile, which were used to identify abnormal values in the beam profiles throughout the monitoring period. These control limits were determined through a systematic evaluation process involving five repeated baseline measurements. The values are expressed as percentages; therefore, they can be directly applied to the baseline measurements of facilities that utilize the same measurement system as ours. Based on the repeated measurements, our system demonstrated measurement uncertainties of 0.65% for the penumbra and 0.3% for the symmetry and flatness, derived from the standard deviations of these measurements. Facilities with measurement systems of higher precision (i.e., smaller uncertainties) would have narrower control limits, whereas those with larger uncertainties would have wider control limits.

**TABLE A1:** Control limits for profile parameters in each orientation.

Orientation	Penumbra (+)	Penumbra (−)	Symmetry	Flatness
Longitudinal	2.4%	3.3%	0.7%	0.7%
Lateral	1.7%	2.1%	0.5%	0.4%
First diagonal	7.5%	8.4%	0.7%	0.6%
Second diagonal	8.0%	7.4%	0.5%	0.6%

*Note*: Penumbra (+) and penumbra (−) denote the positive and negative sides of the penumbra region, respectively.

## SCANNING‐DIRECTION DEPENDENCE IN THE OCTAVIUS DETECTOR

Figure A3 depicts the variation in beam profiles acquired by the Octavius detector array when oriented along the orthogonal (longitudinal and lateral) and diagonal axes. The measurements reveal discrepancies in the profiles for an identical CyberKnife beam direction when the Octavius detector is rotated by 45°. While no substantial differences were observed within the detector's densely populated central region of ± 22.5 mm, the detector response exhibited measurable deviations beyond this range. In this particular instance, the negative side penumbra width measured by the detector in the longitudinal orientation was approximately 4.7 mm, whereas the corresponding measurement in the first diagonal orientation was 5.1 mm. These observations indicate that the discrepancies in penumbra width between orthogonal and diagonal detector channels are more likely a result of the intrinsic properties of the Octavius detector rather than genuine fluctuations in the CyberKnife beam profile.
